# Circulating CD137⁺Treg cells and LOX-1⁺PMN-MDSCs as biomarkers of immunotherapy resistance in (R/M) HNSCC patients

**DOI:** 10.1186/s13046-025-03574-6

**Published:** 2025-12-03

**Authors:** Angela Asquino, Alessio Cirillo, Lidia Strigari, Angelica Pace, Chiara Napoletano, Lucrezia Tuosto, Flavio Valentino, Andrea Ballario, Daniele Santini, Marianna Nuti, Andrea Botticelli, Aurelia Rughetti, Ilaria Grazia Zizzari

**Affiliations:** 1 Laboratory of Tumor Immunology and Cell Therapy, Department of Experimental Medicine, “Sapienza” University of Rome, Viale Regina Elena 324, Rome, Italy; 2 Division of Oncology, Department of Radiological, Oncological and Pathological Science, Policlinico Umberto I, Sapienza University of Rome, Rome, Italy; 3 Department of Medical Physics, IRCCS Azienda Ospedaliero-Universitaria di Bologna, Bologna, Italy; 4 Division of Oncology, Department of Science and Medical-Surgery Biotechnologies, Policlinico Umberto I, Sapienza University of Rome, Rome, Italy

**Keywords:** (R/M) HNSCC, Anti-PD-1 immunotherapy, Immune suppression, CD137^+^ Treg cells, LOX-1⁺PMN-MDSCs, Blood liquid biopsy

## Abstract

**Background:**

Recurrent/metastatic head and neck squamous cell carcinoma ((R/M) HNSCC) represents one of the most aggressive and immunosuppressive cancers. Despite the introduction of immune checkpoint inhibitors (ICIs), only a limited number of patients obtain long-term benefits. In (R/M) HNSCC patients, the antitumor immune response is defective, conferring resistance and promoting tumor progression. Therefore, the identification of novel biomarkers for superior clinical outcomes and easily accessible in standard clinical settings is still an unmet clinical need.

**Methods:**

Blood liquid biopsies obtained from (R/M) HNSCC patients undergoing pembrolizumab therapy (monotherapy or in combination with chemotherapy) were analyzed by flow cytometry to evaluate the levels of circulating immunosuppressive regulatory T cells (Tregs) and myeloid derived suppressor cells (MDSCs), at baseline and during therapy. Correlations between these immunosuppressive immune cell subsets and clinical parameters (clinical response rate, progression-free survival (PFS), overall survival (OS) and performance status (PS)) were performed.

**Results:**

Univariate analysis showed that before therapy, higher circulating levels of both CD137⁺Tregs and LOX-1⁺PMN-MDSCs, identified patients with significantly worse survival. Furthermore, CD137⁺Tregs resulted also positively correlated with worse PS, while high levels of LOX-1⁺PMN-MDSCs negatively affected response to pembrolizumab, with a significant increase in non-responsive patients during therapy. Interestingly, both CD137⁺Tregs as well as LOX-1⁺PMN-MDSCs exerted a higher immunosuppression on T cell proliferation than CD137^−^Tregs and LOX-1⁻PMN-MDSCs, respectively. Multivariate analysis revealed that the circulating LOX-1⁺PMN-MDSC subset resulted as an independent prognostic factor for survival by multivariate analysis, as confirmed in an independent validation cohort.

**Conclusions:**

The levels of blood circulating LOX-1⁺PMN-MDSCs may be proposed as non-invasive biomarkers to predict clinical outcomes of (R/M) HNSCC patients developing resistance to immunotherapy, improving patient selection and suggesting novel personalized therapies.

**Supplementary Information:**

The online version contains supplementary material available at 10.1186/s13046-025-03574-6.

## Background

 The global occurrence of head and neck tumors is the sixth most common type of cancer, with about 900,000 new cases each year [1]. Head and neck squamous cell carcinoma (HNSCC) represents the most common histological type and develops in the mucosal surfaces of oral cavity, sinonasal cavity, pharynx and larynx [2]. Recent progress in therapeutic strategies has only slightly improved the overall prognosis for HNSCC patients, especially for recurrent or metastatic (R/M) HNSCC. Immunotherapy with immune checkpoint inhibitors (ICIs) is the first-line treatment for (R/M) HNSCC patients. To date, pembrolizumab, an anti-PD-1 antibody, is used as first-line therapy in combination with platinum-based chemotherapy and 5-fluorouracil chemotherapy, as established by the Keynote 048 trial [3]. For clinically frail patients with significant comorbidities, pembrolizumab is administered as monotherapy [3]. Until now, the eligibility of HNSCC patients for anti-PD-1 therapy is determined by the combined positive score (CPS), which includes the total number of PD-L1^+^ cells (both tumor and immune cells). The CPS value is also employed as a predictive biomarker [4]: patients with a CPS ≥ 20, and to a lesser extent CPS ≥ 1, have an increased objective response rate and better therapy survival [3, 5–7]. However, despite CPS is required for the administration of immunotherapy and appears to be useful in identifying patients with a greater likelihood of response to pembrolizumab, it is currently deemed insufficient for predicting clinical benefit. The initial response rate to immunotherapy remains still limited in this patient group, highlighting the urgent need to investigate immune evasion mechanisms that induce therapeutic resistance, as well as immune-related biomarkers indicative of tumor progression.

Currently, many candidate biomarkers, including the platelet-to-lymphocyte ratio (PLR), lymphocyte-to-monocyte ratio (LMR), PD-L1 and PD-L2 expression, human papilloma virus (HPV) expression, tumor-infiltrating lymphocyte (TIL) count, and tumor mutational burden (TMB), are being investigated in HNSCC patients [8–10]. However, they have not yet been validated as biomarkers for clinical use due to their dynamic variations during cancer and the availability of material that cannot be analyzed over time. The failure of immunotherapeutic treatment in HNSCC patients appears to be strictly dependent on the highly immunosuppressive burden in the tumor microenvironment (TME) [11, 12], impacting disease recurrence and affecting the overall survival (OS) of HNSCC patients [13]. Among the cells of the immune system, myeloid-derived suppressor cells (MDSCs) and regulatory T cells (Tregs) significantly contribute to the establishment of an immunosuppressive TME in HNSCC [14–16]. MDSCs are pathologically activated immune cells with immunosuppressive activity [17, 18]. According to their origin, they can be subdivided into two distinct subsets: the monocytic (M-MDSC) and the granulocytic (PMN-MDSC) lineages. In particular, the PMN-MDSCs expressing the lectin-type oxidized LDL receptor 1 (LOX-1) appear to be the MDSC subset with the most potent immunosuppressive property. Indeed, several studies have outlined the role of LOX-1⁺PMN-MDSCs in tumor spreading, metastasis and immune suppression [19]. In fact, PMN-MDSCs also enhance tumor angiogenesis through the release of angiogenic factors promoting metastatic progression [20]. Also, regulatory T cells play a crucial role in the inhibition of anti-tumor immune cells and in promoting tumor development and progression. Their intra-tumoral levels are associated with poor survival in several cancers [21], including HNSCC [22]. However, recent studies have shown that high levels of Tregs positively correlate with the survival rate of patients with oral cancer [23–25]. Therefore, the impact of Tregs on the prognosis of patients with HNSCC still remains unclear. In the last years, several studies have been conducted to better define the surface markers of Tregs involved in their suppressive activity [26]. Recently, CD137 (4-1BB) was identified as a signature marker for intratumoral Tregs [27–30]. It was demonstrated that CD137⁺Treg cells exert stronger suppressive effects than CD137⁻Treg cells [25]. CD137 is a costimulatory molecule, member of TNFR family, expressed by activated leukocytes and myeloid cells. It lacks cytoplasmic death domains and therefore does not directly interact with the caspase activation machinery [31–33]. In cancer immunity, effector CD137⁺T cells have emerged as an optimal immune biomarker for defining the response of (R/M) HNSCC patients to pembrolizumab treatment [34] and for predicting the clinical outcome of patients receiving cancer treatments [35, 36]. Although many efforts have been made to identify the immune-molecular patterns characterizing the tumor microenvironment and possibly predictors of therapeutic response, it is difficult to transpose them as useful biomarkers in clinical practice. On the other hand, the availability of biomarkers predictive of clinical response or resistance to immunotherapy, obtained by blood liquid biopsy, is still lacking, especially for (R/M) HNSCC.

In this study we have investigated, for the first time, the role of circulating Treg and MDSC subsets in (R/M) HNSCC patients receiving pembrolizumab treatment. We correlated the levels of circulating Treg cells (total, resting, activated, non-suppressive (ns), and CD137⁺) and MDSCs (LOX-1⁺PMN-MDSCs and M-MDSCs) with clinical parameters, such as Eastern Cooperative Oncology Group performance status (ECOG PS), clinical response after six months of pembrolizumab treatment, progression-free survival (PFS) and overall survival (OS). We identified blood circulating CD137⁺Tregs and LOX-1⁺PMN-MDSCs as novel biomarkers associated with clinical outcomes, potentially useful for improving patient selection and the efficacy of immunotherapy in (R/M) HNSCC patients. CD137^+^Tregs were found to be predictive factors negatively associated with survival, while LOX-1⁺PMN-MDSCs emerged as independent negative prognostic factors related to immunotherapy resistance. The immunosuppressive mechanisms of both CD137⁺Tregs and LOX-1⁺PMN-MDSCs were investigated in *vitro*. Furthermore, the role of LOX-1⁺PMN-MDSCs as predictor of immunotherapy resistance, was confirmed in a validation cohort. The results highlight the potential of blood circulating LOX-1⁺PMN-MDSCs as non-invasive biomarkers for predicting immunotherapy resistance in (R/M) HNSCC patients.

## Methods

### Patients

From February 2021 to December 2024, eighty patients with a confirmed diagnosis of (R/M) HNSCC receiving anti-PD-1 treatment were prospectively enrolled at the Medical Oncology Department of Policlinico Umberto I Hospital-Sapienza University. Patients were treated either with pembrolizumab monotherapy or with pembrolizumab in combination with 5-fluorouracil and the platinum drug of choice among cisplatin and carboplatin (pembrolizumab-based chemoimmunotherapy) until disease progression or unacceptable toxicity. The choice of the specific treatment regimen and dosages is based on the KEYNOTE-048 trial [3]. Patients were divided into two cohorts: the discovery cohort, which included the first 40 patients enrolled and the validation cohort, which included the subsequent 40 patients. The inclusion criteria used in this study were as follows: age >18 years; histologically documented diagnosis of HNSCC of the oral cavity, oropharynx, larynx, or nasopharynx; combined positive score (CPS) ≥ 1; and ECOG PS score between 0 and 2. CPS was defined as the number of PD-L1 positive cells (tumor cells, lymphocytes, and macrophages) divided by the total number of viable tumor cells multiplied by 100. The exclusion criteria were autoimmune disease, systemic immunosuppression and any significant comorbidity. PFS, OS and the clinical benefit rate (CBR) were evaluated. PFS was defined as the time from the start of immunotherapy until the first documented tumor progression or death from any cause. OS was defined as the time between the beginning of immunotherapy and death from any cause. Responsive (patients with a complete or partial response and stable disease) and non-responsive (progressors) patients were classified after 6 months of therapy using the CBR. The study was conducted following the Declaration of Helsinki and good clinical practice guidelines. All patients provided signed informed consent (RIF. CE: 4181, Policlinico Umberto I, Sapienza University of Rome, Italy).

### Peripheral blood mononuclear cell and Sera collection

Peripheral blood mononuclear cells (PBMCs) derived from blood samples of (R/M) HNSCC patients were isolated using Ficoll Hypaque density gradient (Lympholyte-H, Cedarlane, Burlington, VT, Canada). PBMCs were isolated both at baseline (T0), just before the beginning of immunotherapy and after one cycle of anti-PD-1 treatment (T1) for the discovery cohort, while only at baseline for the validation cohort. Serum was concurrently purified by centrifugation at 1.800 rpm for 10 min using the BD Vacutainer Plus Plastic Serum tubes (Becton Dickinson, Franklin Lakes, New Jersey, U.S.). PBMCs and patients’ sera were cryopreserved until use.

### Immunophenotyping

PBMCs were characterized by multiparametric flow cytometry. The following monoclonal antibodies (mAbs) were used to assess the different circulating immunosuppressive cells: anti-CD3 BV510 (HIT3A clone), anti-CD4 APC-H7 (RPA-T4 clone), CD45RA BB515 (HI100 clone), CD137 BV421 (4B4-1 clone) (all from BD Biosciences, San Jose, CA, USA), CD25 PE (MA251clone) (BioLegend, San Diego, CA, USA), FOXP3 APC (PCH101 clone) (Thermo Fisher Scientific, Waltham, MA, USA) to analyze Treg cells; the analysis of CD137⁺Treg cells was performed in 35 patients for the discovery cohort. The differential combined expression of CD45RA and FOXP3 was used to discriminate different subsets of Tregs: active (CD4⁺FOXP3^high^CD45RA⁻), resting (CD4⁺FOXP3^low^CD45RA⁺) and non-suppressive (CD4⁺FOXP3^low^CD45RA⁻). MDSCs were analyzed in freshly isolated PBMCs from 70 patients (30 from the discovery cohort and 40 from the validation cohort) utilizing the following mAbs: HLA-DR FITC (L243 clone), CD45 AF700 (2D1 clone), LOX1 PE (15C4 clone), CD14 BB700 (MφP9 clone), and CD15 APC (HI98 clone) (all from Biolegend) and CD66b Pe-Cy7 (DREG-5 clone, BD Biosciences, San Jose, CA, USA). Specifically, the following MDSC subsets were identified: LOX-1⁺PMN-MDSCs (CD66b⁺ HLA-DR⁻ LOX-1⁺) and M-MDSCs (CD66b⁻CD14⁺ HLADR⁻). Fluorescence minus one (FMO) was used as a negative control. The Zombie Aqua Fixable Viability Kit (BioLegend) was used to identify live cells, while the Foxp3/Transcription Factor Staining Buffer Set (Invitrogen, Waltham, MA) was used for intracellular staining. To analyze the intracellular expression of Arginase-1 (ARG1), PBMCs were first stained with surface antibodies: anti-CD66b Pe-Cy7 (DREG-5 clone, BD Biosciences, San Jose, CA), CD45 AF700 (2D1 clone), HLA-DR APC-Cy7 (L243 clone) and LOX-1 PE (15C4 clone) (all from Biolegend, San Diego, CA, USA); CD15 PB (80H5 clone) and CD14 ECD (RM052 clone) (both from Beckman Coulter). Cell viability was assessed using the LIVE/DEAD™ Fixable Yellow Dead Cell Stain Kit (Thermo Fisher Scientific). Cells were then fixed and permeabilized with Cytofix/Cytoperm (BD Biosciences, San Jose, CA) for 20 min at 4 °C. Intracellular staining was then performed with anti-ARG1 APC (14D2C43 clone, Biolegend) for 30 min at 4 °C. FMO for ARG1 was used as negative control. Results of ARG1 were expressed as mean fluorescence intensity (MFI). The samples were acquired by a DxFLEX Flow Cytometer (Beckman Coulter, Brea, CA) and analyzed by FlowJo analysis software (version 10.8.8, Becton Dickinson).

### Detection of intracellular ROS

Intracellular reactive oxygen species (ROS) levels were measured using the 2’,7’-dichlorofluorescein diacetate (DCFDA) staining (Abcam, AB113851). PMBCs were stained with the following antibodies: anti-CD66b Pe-Cy7 (DREG-5 clone, BD Biosciences, San Jose, CA), CD45 AF700 (2D1 clone), HLA-DR APC-Cy7 (L243 clone) and LOX-1 PE (15C4 clone) (all from Biolegend); CD15 PB (80H5 clone) and CD14 ECD (RM052 clone) (both from Beckman Coulter), LIVE/DEAD™ Fixable Yellow Dead Cell Stain Kit (Thermo Fisher Scientific), to identify live cells, and were incubated with 20 µM DCFDA at 37° for 30 min. ROS levels were analyzed by the DxFLEX Flow Cytometer (Beckman Coulter, Brea, CA) and analyzed by FlowJo analysis software (version 10.8.8, Becton Dickinson). Results were expressed as MFI.

### Isolation of CD137^+^Tregs from peripheral blood of (R/M) HNSCC patients and suppression assay

Treg cells were isolated from PBMCs of (R/M) HNSCC patients employing the human CD4^+^CD127^low^CD25^+^Regulatory T Cells kit (StemCell Technologies) following the manufacturer’s protocol. The purified Tregs were then stained with anti-CD137-PE mAb (Thermo Fisher Scientific) and separated into CD137^+^ and CD137^−^ fractions using an EasySep™ Release Human PE Positive Selection Kit and an EasySep™Magnet (both from StemCell Technologies). CD4^+^CD25^−^ T cells were labeled with 5,6-carboxyfluorescein diacetate, succinimidyl ester (CFSE) (1µM, Invitrogen, Carlsbad, CA) and cultured in Roswell Park Memorial Institute Medium (RPMI)−1640 supplemented with L-glutamine (Sigma-Aldrich, G7513), penicillin 100 U/mL, streptomycin 100 mg/mL (Penicillin-Streptomycin solution, Sigma Aldrich, P0781) and 5% heat-inactivated fetal calf serum (FCS; Merck KGaA, Darmstadt, Germany). CD4^+^CD25^−^ T cells and CD137^+^ or CD137^−^ Treg cells, were cultured alone or co-cultured (Tregs: CD4^+^CD25^−^ T cells, 1:2) with anti-CD3 (1 µg/mL, OKT3 clone) and anti-CD28 (1 µg/mL, CD28.2 clone) (both from Invitrogen, Waltham, MA) antibodies for 4 days at 37 °C. After 4 days, cell culture supernatants were collected for cytokine analysis interleukin (IL)−10 and transforming growth factor β (TGF-β)). Cells were stained for flow cytometry analysis using anti-CD4 PB (13B8.2 clone) (Beckman Coulter, Brea, CA), CD127 Pe-Cy7 (eBioRDR5 clone) (Thermo Fisher Scientific), CD137 APC (4B4-1 clone) (BD Biosciences, San Jose, CA) along with LIVE/DEAD™ Fixable Yellow Dead Cell Stain Kit (Thermo Fisher Scientific). T cell proliferation was assessed by CFSE dilution using a DxFLEX Flow Cytometer (Beckman Coulter, Brea, CA) and FlowJo analysis software (version 10.8.8, Becton Dickinson). The proliferation of CD4⁺CD25⁻ T cells cultured alone was set as 100%.

### Isolation of LOX-1⁺PMN-MDSCs from peripheral blood and immune suppression assay

Polymorphonuclear neutrophils (PMNs) were isolated from peripheral blood of (R/M) HNSCC patients using the EasySep™ Human Neutrophil Isolation Kit (StemCell Technologies) according to the manufacturer’s protocol. Isolated cells were then labeled with anti-LOX-1-PE mAb (BioLegend) and separated into LOX-1^+^ and LOX-1^−^ populations using EasySep™ Release Human PE Positive Selection Kit and EasySep™Magnet (both from StemCell Technologies) [20]. Concurrently, T lymphocytes were isolated from PBMCs of the same patients using the EasySep™ Human T Cell Isolation Kit (StemCell Technologies) and labeled with 1µM of CFSE (Invitrogen, Carlsbad, CA). T cells were cultured in RPMI-1640 supplemented with L-glutamine (Sigma-Aldrich, G7513), penicillin 100 U/mL, streptomycin 100 mg/mL (Penicillin-Streptomycin solution, Sigma Aldrich, P0781) and 5% FCS (Merck KGaA, Darmstadt, Germany). Cells were seeded in 96-well plates pre-coated with 1 µg/mL anti-CD3 (OKT3 clone) and 1 µg/mL anti-CD28 beads (CD28.2 clone) (both from Invitrogen, Waltham, MA). T cell were added to the LOX-1⁺ or LOX-1⁻PMN-MDSCs at PMN-MDSC: T cell ratios of 1:1, 1:2. After 4 days of co-culture, supernatants were collected to determine Arginase-1 production while cells were stained for flow cytometry analysis using anti-CD3 KRO (UCHT1 clone), CD4 PB (13B8.2 clone) (both from Beckman Coulter) along with LIVE/DEAD™ Fixable Yellow Dead Cell Stain Kit (Thermo Fisher Scientific). T cell proliferation was assessed by CFSE dilution using a DxFLEX Flow Cytometer (Beckman Coulter, Brea, CA) and FlowJo analysis software (version 10.8.8, Becton Dickinson). The proliferation of T cells cultured alone was defined as 100%.

### IL-10, TGF-β and arginase-1 ELISA assay

The concentration of IL-10 and TGF- β released in the supernatants of Tregs/CD4^+^CD25^−^ T cells co-culture (at a ratio of 1:2) was measured using the Human IL-10 ELISA Kit and the Human TGF β −1 ELISA Kit, respectively, following the manufacturer’s instructions. Arginase-1 levels were quantified in the supernatants of PMN-MDSC/T cell co-cultures (at a 1:2 ratio) and in the sera of (R/M) HNSCC patients using the Human Arginase-1 ELISA Kit, according to the manufacturer’s instructions. All ELISA kits were purchased from Invitrogen, Waltham, MA.

### Statistical analysis

Discrete variables were presented in a descriptive table as absolute numbers and percentages, while continuous variables were summarized using means, ranges, and interquartile ranges. The database was divided into a discovery and a validation cohort (patients 1–40 and patients 41–80, respectively). The impact of circulating immunosuppressive cells on OS and PFS after immunotherapy was analyzed by both univariate and multivariate analyses (UVA and MVA, respectively) using the discovery dataset. The OS and PFS of patients with UVA were analyzed using the Kaplan–Meier method and log-rank tests. The cut-off used to stratify patients for OS and PFS analysis was set at the median percentage value for each immunosuppressive cell subset. Prognostic clinicopathological variables of potential relevance in the univariate analysis, corresponding to a cut-off of *p* < 0.05 at the UVA, were included in the OS or PFS MVA analysis. The proportional hazards assumption was tested using Schoenfeld residuals. The prognostic ability of biomarkers identified using the discovery dataset was assessed in the validation one. *p* < 0.05 was considered to indicate statistical significance. The cut-off statistical analyses were performed using R package software.

## Results

### Patient characteristics

Eighty patients with (R/M) HNSCC who received anti-PD-1 treatment were enrolled in this study (40 for discovery cohort and 40 for validation cohort, Table 1). Patients belonging to the discovery cohort had a CPS ≥ 1 and received pembrolizumab as monotherapy (62.5%) or pembrolizumab plus chemotherapy (37.5%). Before starting the anti-PD-1 treatment, 57.5% of patients had a PS = 1, and 22.5% had a PS = 2. The remaining 20% of patients had a PS = 0. The primary tumor site in most patients was the oral cavity (50%), followed by the larynx (30%) and oropharynx (20%). A CPS > 20 was observed in 60% of the tumor samples. Most patients were current smokers (42%) or previous smokers (38%), and 70% consumed moderate amounts of alcohol. The CBR was used to classify responsive (R) (42.5%) and non-responsive (NR) (57.5%) patients to pembrolizumab treatment. The median PFS and OS were 3 and 7 months, respectively. All patients belonging to the validation cohort had CPS ≥ 1 and were treated with an anti-PD-1 agent as monotherapy (15%) or in combination with chemotherapy (85%). Regarding PS, 67.5% of patients of the validation cohort were classified as PS = 1, 22.5% as PS = 2 and 10% were scored as PS = 0. Clinical response rate was used to classify R (25%) and NR (75%) to anti-PD-1 treatment. The median PFS and OS, for the validation cohort, were 3 and 6.5 months, respectively.

**Table 1.** Patients‘characteristics

Clinical parameters	Discovery cohort	Validation cohort
	(no. of patients/%)	(no. of patients/%)
Total	40 (100)	40 (100)
**Sex**		
Male	32 (80)	25 (62.5)
Female	8 (20)	15 (37.5)
**Age**		
Median range	69.3	69
* ≤6*5	16 (40)	17 (42.5)
> 65	24 (60)	23 (57.5)
**EOCG Performance Status**		
0	8 (20)	4 (10)
1	23 (57.5)	27 (67.5)
2	9 (22.5)	9 (22.5)
**Primary tumour site**		
Oral cavity	20 (50)	33 (82.5)
Larynx	12 (30)	2 (5)
Oropharynx	8 (20)	5 (12.5)
**CPS**		
*≥ 1*	40 (100)	40 (100)
> 20	24 (60)	25 (62.5)
**Therapy for metastatic disease**		
Pembrolizumab	25 (62.5)	6 (15)
Pembrolizumab + CHT	15 (37.5)	34 (85)
**HPV** **status** ^**a**^		
Negative	5 (62.5)	2 (40)
Positive	1 (12.5)	1 (20)
ND	2 (25)	2 (40)
**Smoking status**		
Current smoker	17 (42)	13 (32.5)
Previous smoker	15 (38)	12 (30)
Non-smoker	8 (20)	15 (37.5)
**Alcohol consumption**		
Yes (moderate/high)	28 (70)	8 (20)
No	12 (30)	32 (80)
**Response to treatment**		
Yes	17 (42.5)	10 (25)
No	23 (57.5)	30 (75)

*Abbreviations: ND* Not determined

^a^(Evaluated only on oropharynx)

### Circulating CD137^+^Treg cells are a negative predictive biomarker of survival in (R/M) HNSCC patients treated with anti-PD-1 therapy

**Fig. 1:**
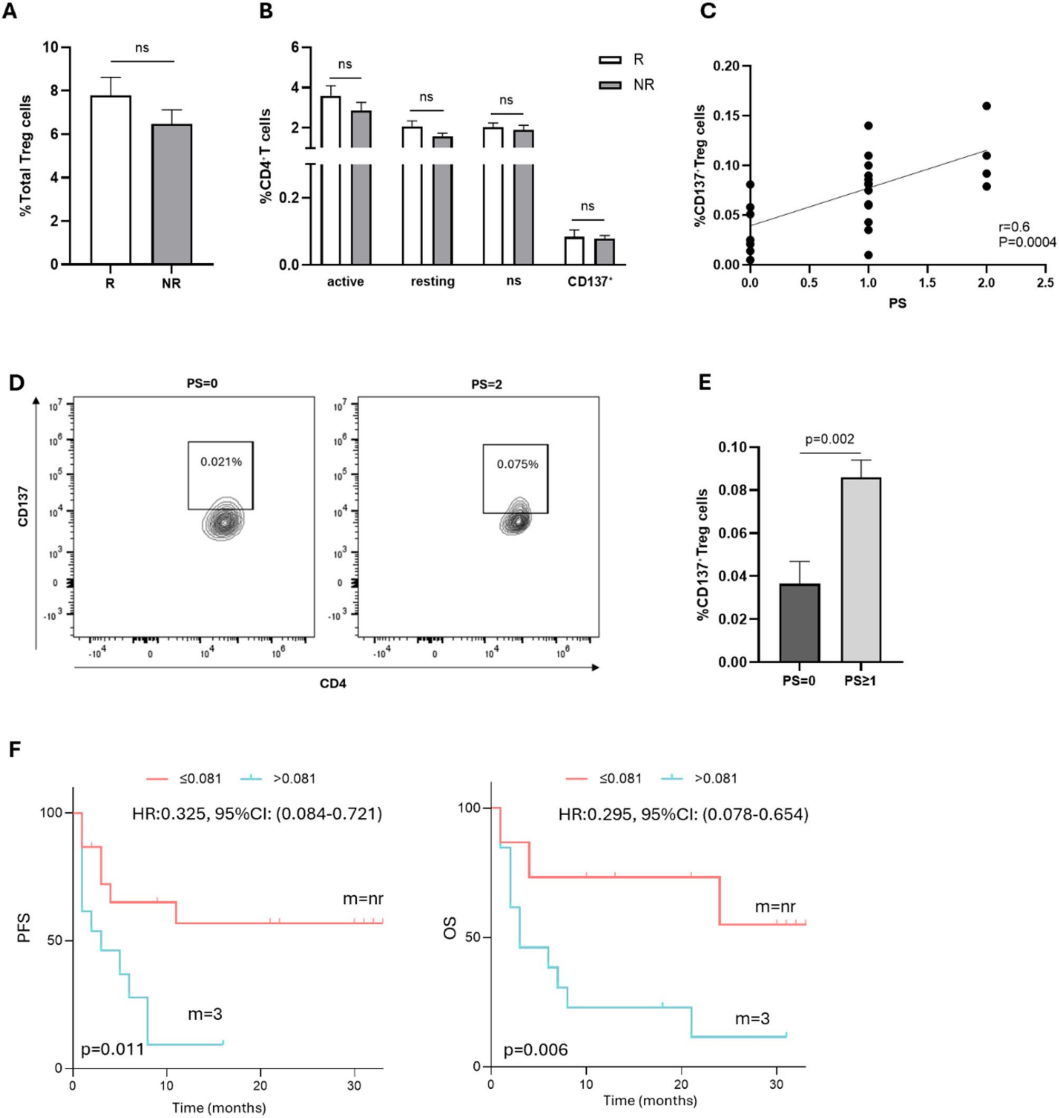
Correlation of circulating Treg cells with clinical parameters in (R/M) HNSCC patients undergoing immunotherapy. **A** Histogram showing the percentage of circulating total regulatory T (Treg) cells at baseline in patients with (R/M) HNSCC classified as responsive (R) or non-responsive (NR) to immunotherapy. The data are shown as the mean ± standard error of the mean (SEM). **B** The histogram displays the levels of different Treg subsets (active, resting, non-suppressive (ns) and CD137^+^) in R and NR patients before the beginning of immunotherapy. The data are shown as the mean ± SEM. **C** Correlation between the percentage of CD137⁺Treg cells and performance status (PS) in (R/M) HNSCC patients treated with pembrolizumab. **D** Cytofluorometric analysis of CD137⁺Treg cells at baseline in a (R/M) HNSCC representative patient with PS = 0 and one patient with PS = 2, receiving anti-PD-1 therapy. **E** Histogram representing the levels of circulating CD137⁺Treg cells in patients with PS = 0 and PS ≥ 1. Patients with a worse clinical status at baseline, PS ≥ 1, had significantly greater levels of CD137⁺Treg cells than patients with PS = 0. The results are shown as the mean values ± SEM. **F** Kaplan‒Meier curves of PFS and OS were generated, and 0.081% (the median value of CD137⁺ Treg cells) was used as the cut-off. Patients with a percentage ≤ 0.081% had significantly longer survival. A long-rank test was used to analyze the differences between the two groups. m = months, nr = not yet reached, p values ≤ 0.05 were considered significant

To evaluate the impact of circulating immunosuppressive cells in (R/M) HNSCC patients, the levels of Treg subsets (total, resting, active, non-suppressive (ns) and CD137⁺) were evaluated in the blood of patients before the beginning of immunotherapy (T0) by flow cytometry. As shown in Fig. 1A, the percentage of total Treg cells did not affect the response to pembrolizumab treatment at baseline, as well as the frequency of different Treg subsets (resting, active, ns, and CD137⁺) (Fig. 1B), indicating that at baseline, Treg cells do not influence the response to treatment in (R/M) HNSCC patients’ setting. Interestingly, when we correlated Treg cells with other clinical parameters, the CD137⁺Tregs emerged as the only Tregs subset associated with clinical status and survival. In fact, the level of CD137⁺Treg cells resulted positively correlated with the PS of (R/M) HNSCC patients (*p* = 0.0004 and *r* = 0.6) (Fig. 1C). Figure 1D shows staining profiles of CD137⁺Tregs in the blood of one representative (R/M) HNSCC patient scored as PS = 0 and one scored as PS = 2. Patients with a worse clinical status at baseline (PS ≥ 1) had higher levels of CD137⁺Treg cells than patients with PS = 0 (PS = 0 vs. PS ≥ 1: 0.036%±0.010 for CD137⁺Tregs vs. 0.086%±0.008, *p* = 0.002) (Fig. 1E). Moreover, patients with high blood circulating levels of CD137⁺Tregs (> 0.081%) had poor survival in terms of both PFS (CD137⁺Tregs > 0.081% vs. CD137⁺Tregs ≤ 0.081%: median survival 3 months vs. nr (not yet reached), HR: 0.325, 95% CI (0.084–0.721), *p* = 0.011) and OS (CD137⁺Tregs 0.081% vs. CD137⁺Tregs ≤ 0.081%: median survival 3 months vs. nr, HR:0.295, 95% CI (0.078–0.654), *p* = 0.006) (Fig. 1F; Table 2, Univariate analysis). No correlation between PS and survival was found for total Tregs (Additional File 1). These data highlight the predictive role of CD137⁺Tregs in the survival of (R/M) HNSCC patients receiving immunotherapy.

### CD137^+^Tregs isolated from (R/M) HNSCC patients exhibit a pronounced immune suppressive capacity

**Fig. 2:**
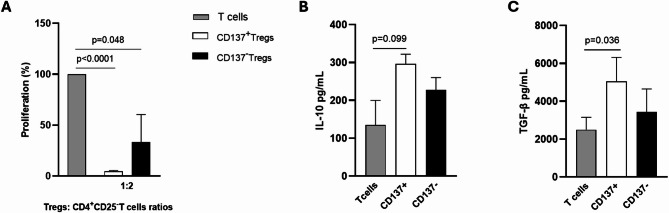
Immune suppression activity of CD137^+^Tregs derived from peripheral blood of (R/M) HNSCC patients. **A** Suppression assay of CD137⁺ (white column) and CD137⁻Tregs (black column) isolated from (R/M) HNSCC, co-cultured for 4 days with autologous CD4^+^CD25^−^T cells (T cells, grey column). Data indicate the mean ± SEM of three independent experiments. **B** Levels of IL-10 in the supernatants of Tregs/CD4^+^CD25^−^T cells at ratio 1:2 by ELISA assay. **C** Release of TGF-β in the supernatants of Tregs/CD4^+^CD25^−^T cells at ratio 1:2 by ELISA assay. p values ≤ 0.05 were considered significant

To assess the immunosuppressive capacity of circulating CD137^+^ Tregs, CD4^+^CD25^−^ T cells and CD137^+^ and CD137^−^ Tregs were isolated from PBMCs of (R/M) HNSCC patients. As expected, CD4^+^CD25^−^ T cells alone, stimulated with anti-CD3 and anti-CD28, exhibited strong proliferative capacity (Fig. 2A). The proliferation resulted inhibited when T cells were co-cultured with CD137^+^ and CD137^−^ Tregs (ratio 1:2). However, data showed that CD137^+^Tregs exhibit stronger suppressive activity than CD137^−^Tregs (*p* < 0.001 vs. *p* = 0.048; Fig. 2A). Moreover, to investigate the mechanism underlying CD137^+^Treg-mediated suppression, the levels of IL-10 and TGF-β released in the co-culture supernatant were evaluated. The results showed that CD137^+^Tregs released higher levels of IL-10, although this increase was a trend (*p* = 0.099, Fig. 2B) and a significantly greater amount of TGF-β (*p* = 0.036, Fig. 2C) compared to CD137^−^Tregs, suggesting an enhanced immune-suppressive profile of CD137^+^Tregs.

### Peripheral LOX-1⁺PMN-MDSCs impact the response to immunotherapy and the survival of (R/M) HNSCC patients

**Fig. 3:**
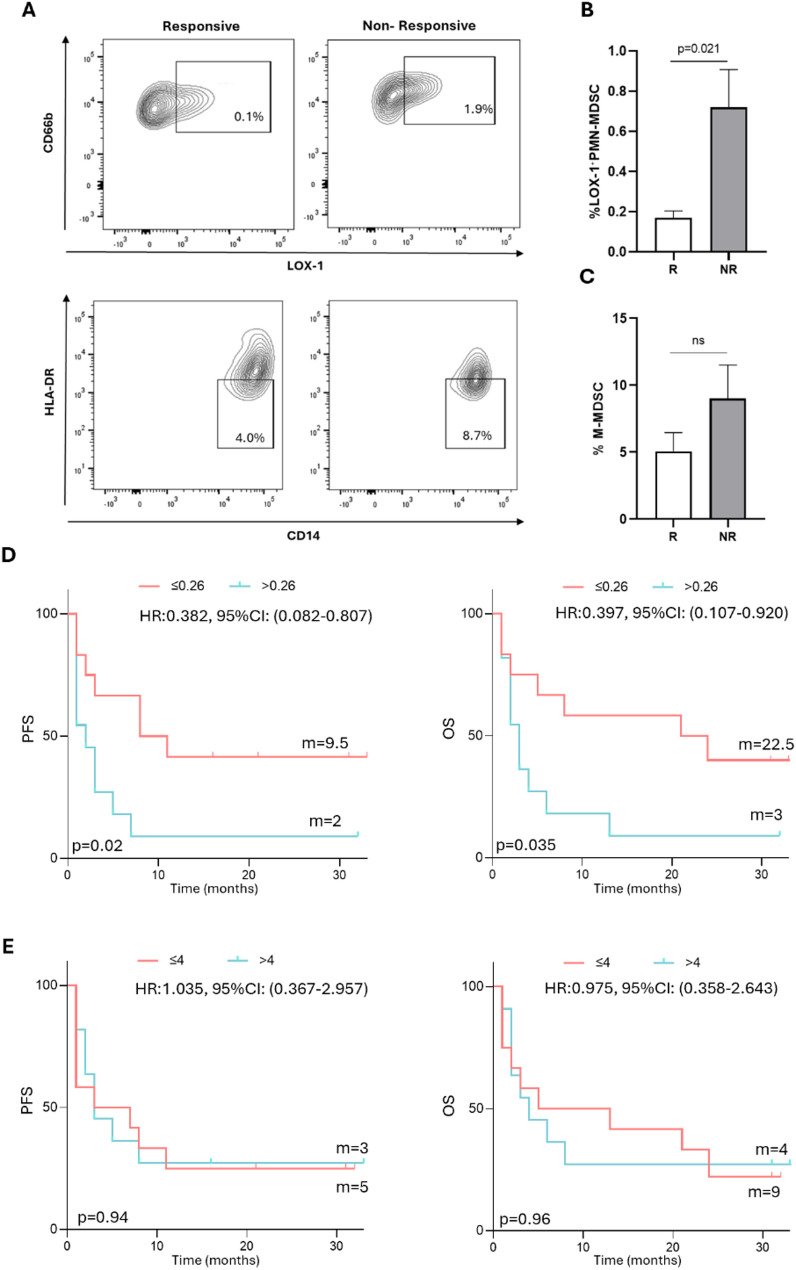
Modulation of circulating MDSCs in responsive and non-responsive patients and correlation with survival. **A** Cytofluorometric analysis of LOX-1⁺PMN-MDSCs and M-MDSCs at baseline in representative responsive (R) and non-responsive (NR) (R/M) HNSCC patients receiving immunotherapy. LOX-1⁺PMN-MDSCs were identified as CD66b⁺ HLA-DR⁻ LOX-1⁺, while M-MDSCs were identified as CD66b⁻CD14⁺ HLA-DR⁻. **B** Histogram showing the percentage of circulating LOX-1⁺PMN-MDSCs ± SEM at baseline in (R/M) HNSCC patients treated with pembrolizumab. The percentage of LOX-1⁺PMN-MDSCs in NR patients was significantly higher than those in R patients. **C** The percentage of M-MDSCs ± SEM in R and NR patients is shown as a histogram. **D** Kaplan‒Meier curves of PFS and OS were generated using a cut-off value of 0.26%, the median percentage of circulating LOX-1⁺PMN-MDSCs. Survival analysis revealed that patients with a percentage ≤ 0.26% had longer PFS and OS. **E** Kaplan‒Meier curves for PFS and OS were generated, and the median value of 4% was used as the cut-off for M-MDSCs at baseline. A long-rank test was used to analyze the differences between the two groups. m = months, p values ≤ 0.05 were considered significant

To evaluate the contribution of blood circulating MDSCs to the clinical outcome of (R/M) HNSCC patients, the levels of LOX-1⁺PMN-MDSCs and M-MDSCs were freshly analyzed at baseline (T0). Figure 3A shows staining profiles of LOX-1⁺PMN-MDSCs and M-MDSCs in the blood of a responsive and a non-responsive (R/M) HNSCC patient. The results revealed that the level of LOX-1⁺PMN-MDSCs was lower in responsive patients than in non-responsive patients (R vs. NR: 0.168%±0.035 vs. 0.719%±0.187, *p* = 0.021; Fig. 3B), while no significant difference was observed in the levels of M-MDSCs (R vs. NR: 5.056%±1.40 vs. 9.002%±2.509, *p* = 0.220; Fig. 3C). These data suggest that among myeloid subpopulations, only the LOX-1⁺PMN-MDSCs significantly impact the response to immunotherapy in this patients’ setting. Interestingly, the levels of LOX-1⁺PMN-MDSCs were also negatively associated with survival. Indeed, patients with high levels of LOX-1⁺PMN-MDSCs (> 0.26%) at baseline, had worse PFS and OS (PFS: LOX-1⁺PMN-MDSCs > 0.26% vs. LOX-1⁺PMN-MDSCs ≤ 0.26%: median survival 2 vs. 9.5 months, HR:0.382, 95% CI (0.082–0.807), *p* = 0.020; OS: LOX-1⁺PMN-MDSCs > 0.26% vs. LOX-1⁺PMN-MDSCs ≤ 0.26%: median survival 3 months vs. 22.5 months, HR:0.397, 95% CI (0.107–0.920), *p* = 0.035) (Fig. 3D). No significant correlation was observed between M-MDSCs and PFS or OS, respectively (Fig. 3E). These results indicate that circulating LOX-1⁺PMN-MDSCs may represent a predictive biomarker for both response and survival in (R/M) HNSCC patients treated with immunotherapy.

### LOX-1⁺PMN-MDSCs suppress autologous T cells in (R/M) HNSCC patients

**Fig. 4:**
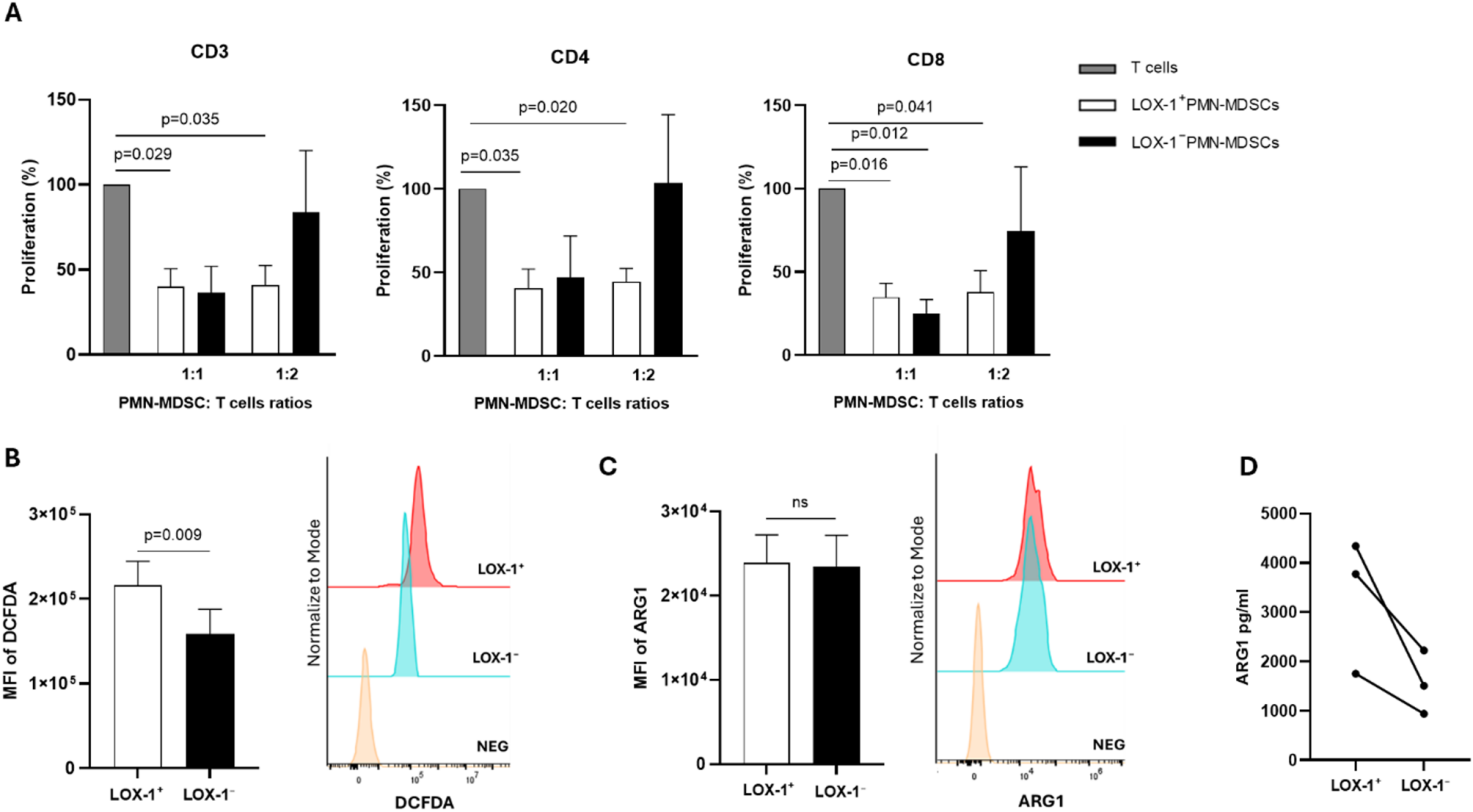
Immune suppression activity of LOX-1⁺PMN-MDSCs derived from peripheral blood of (R/M) HNSCC patients. **A** Suppression assay of LOX-1⁺ (white column) and LOX-1⁻ PMN-MDSCs (black column) isolated from the same patient with (R/M) HNSCC, co-cultured for 4 days with autologous T cells. The data indicate the mean ± SEM of three independent experiments. **B** Intracellular ROS level in LOX-1⁺ (white) or LOX-1⁻ (black) PMN-MDSCs detected by DCFDA staining, Left: Histogram showing the mean fluorescence intensity (MFI) of DCFDA LOX-1⁺ and LOX-1⁻ PMN-MDSC, Right: representative flow cytometry data. **C** Expression of intracellular ARG1 in LOX-1⁺ (white) or LOX-1⁻ (black) PMN-MDSC. Left: Histogram showing the MFI of ARG1 in LOX-1⁺ and LOX-1⁻ PMN-MDSC, Right: representative flow cytometry data. **D** Production of ARG1 in supernatants of LOX-1⁺ and LOX-1⁻ PMN-MDSC/T cells at ratio 1:2 by ELISA assay.p values ≤ 0.05 were considered significant

In order to evaluate the immune suppressive capacity of circulating LOX-1⁺PMN-MDSCs, T cells, LOX-1⁺ and LOX-1⁻PMN-MDSCs were isolated from peripheral blood of (R/M) HNSCC patients. CFSE-labeled T cells were stimulated with anti-CD3/CD28 and co-cultured with LOX-1⁺ or LOX-1⁻PMN-MDSCs at the indicated ratios (Fig. 4). The addition of LOX-1⁺PMN-MDSCs significantly reduced T cell proliferation capacity, as observed for CD3⁺ T cells (ratio PMN-MDSC/T cells 1:1, *p* = 0.029; ratio 1:2, *p* = 0.035), CD4⁺ (ratio PMN-MDSC/T cells 1:1, *p* = 0.035; ratio PMN-MDSC/T cells 1:2, *p* = 0.020) and CD8⁺T cell subsets (ratio PMN-MDSC/T cells 1:1, *p* = 0.016; ratio PMN-MDSC/T cells 1:2, *p* = 0.041), compared to LOX-1⁻PMN-MDSCs (Fig. 4A). These data demonstrated the immune-suppressive capacity of circulating LOX-1⁺PMN-MDSCs, confirming the role of LOX-1⁺PMN-MDSCs in conferring stronger suppressive activity to PMN-MDSCs. Moreover, to investigate the mechanisms involved in LOX-1⁺PMN-MDSCs mediated T cell suppression, the intracellular levels of ROS and Arginase-1 (ARG1) were evaluated. These molecules represent the two main factors involved in MDSC immune suppression capacity. Firstly, the overall PMN-MDSC subset did express significantly higher levels of ARG1 than M-MDSCs (Additional File 2, Panel A), while no significant difference was observed for intracellular ROS production (Additional File 2, Panel B). However, when LOX-1⁺PMN-MDSCs and LOX-1⁻PMN-MDSCs were considered, intracellular ROS levels were higher in LOX-1⁺PMN-MDSCs than LOX-1⁻PMN-MDSCs (*p* = 0.009; Fig. 4B). Interestingly, while no difference in the intracellular ARG1 expression was observed between LOX-1⁺ and LOX-1⁻PMN-MDSCs (*p* = 0.719, Fig. 4C**)**, the secreted ARG1 was higher in LOX-1⁺PMN-MDSCs/T cells co-culture than LOX-1⁻PMN-MDSCs/T cells (Fig. 4D). These results suggest that ARG1 and ROS production may play an important role in the suppressive function of LOX-1⁺PMN-MDSCs in (R/M) HNSCC patients undergoing immunotherapy.

### LOX-1⁺PMN-MDSCs are an independent prognostic factor for PFS and OS in (R/M) HNSCC patients

The biological and clinical parameters of (R/M) HNSCC patients examined by UVA to predict survival are summarized in Table 2. In addition to Tregs and MDSCs described above, this analysis showed that among the clinical parameters, PS is the only parameter associated with prolonged PFS and OS, in accordance with our previous results [34, 37]. Specifically, patients with a PS = 0 had longer PFS (HR: 0.331, 95% CI (0.154–0.946), *p* = 0.038) and OS (HR: 0.292, 95% CI (0.159–0.874), *p* = 0.023) than patients with a PS ≥ 1 (Additional File 3). As shown in Table 2, MVA incorporating both biological (i.e., CD137⁺Treg cells and LOX-1⁺PMN-MDSCs) and clinical parameters (i.e., ECOG Performance Status) identified LOX-1⁺PMN-MDSCs as the only independent prognostic factor for both PFS and OS. Specifically, higher levels of LOX-1⁺PMN-MDSCs were significantly associated with worse PFS (HR = 3.422, 95% CI (1.312–2.148), *p* = 0.009) and OS (HR = 2.901, 95% CI (1.187–1.943), *p* = 0.018), underscoring the pivotal role of this immunosuppressive myeloid population in influencing the clinical outcome of (R/M) HNSCC patients undergoing anti–PD-1 immunotherapy. Assessment of the proportional hazards’ assumption using Schoenfeld residuals indicated no violation for either PFS (variable *p* = 0.52; global *p* = 0.52, data not shown) or OS (variable *p* = 0.20; global *p* = 0.20, data not shown). Therefore, the Cox proportional hazards models were considered valid, supporting the robustness of the observed associations.

**Table 2:** Predictive and prognostic factors for progression free survival and overall survival evaluated in HNSCC patients​

Variables​	Univariate analysis​			Multivariate analysis​
**PFS**	**HR (95%CI)​**	***p*** **value​**	**HR (95%CI)​**	***p*** **value​**
Previous Therapy​	1.023 (0.427–2.470)	0.952		
**Performance Status** **(0 vs.≥1)**​	**0.331** **(0.154–0.946)**	**0.038**		
Treg cells (≤ 6.81% vs. >6.81%)​	1.702 (0.798–4.431)	0.149		
Active Treg cells (≤ 3.10% vs. >3.10%)​	1.335 (0.600–3.281)	0.435		
Resting Treg cells (≤ 1.71% vs. >1.71%)​	0.962 (0.414–2.209)	0.917		
Non-suppressive Treg cells (≤ 1.76% vs. >1.76%)​	0.852 (0.361–1.927)	0.671		
**CD137⁺Treg** **cells** **(≤ 0.081%** **vs.** **>0.081%)​**	**0.325** **(0.084–0.721)**	**0.011**		
**LOX-1⁺PMN-MDSCs** **(≤ 0.26%** **vs. >0.26%**)​	**0**.**382 (0.082–0.807)**	**0.020**	**3.422** (**0.312–2.148)**	**0.009**
M-MDSCs (≤ 4% vs. >4%)​	1.035 (0.367–2.957)	0.938		
**OS**				
Previous Therapy​	1.041 (0.448–2.436)	0.918		
**Performance Status** **(0** **vs**.**≥1)**​	**0.292** **(0.159–0.874**)	**0.023**		
Treg cells (≤ 6.81% vs. >6.81%)​	1.609 (0.739–3.829)	0.215		
Active Treg cells (≤ 3.10% vs. >3.10%)​	1.230 (0.554–2.812)	0.593		
Resting Treg cells (≤ 1.71% vs. >1.71%)​	0.997 (0.442–2.250)	0.994		
Non-suppressive Treg cells (≤ 1.76% vs. >1.76%)​	0.733 (0.318–1.617)	0.424		
**CD137⁺Treg** **cells** **(≤ 0.081%** **vs.** **>0.081%)​**	**0.295** **(0.078–0.654)**	**0.006**		
**LOX-1⁺PMN-MDSCs (≤ 0.26%** **vs.** **>0.26%)**​	**0.397** **(0.107–0.920)**	**0.035**	**2.901** **(0.187–1.943)**	**0.018**
M-MDSCs (≤ 4% vs. >4%)​	0.975 (0.358–2.643)	0.956		

Performance status, CD137⁺ Treg cells and LOX1⁺ PMN-MDSCs, which result significant in the univariate analysis, are marked and are the only ones that were retained relevant for the MVA

### Validation of LOX-1⁺PMN-MDSCs as negative prognostic factor in a prospective cohort of (R/M) HNSCC patients

**Fig. 5:**
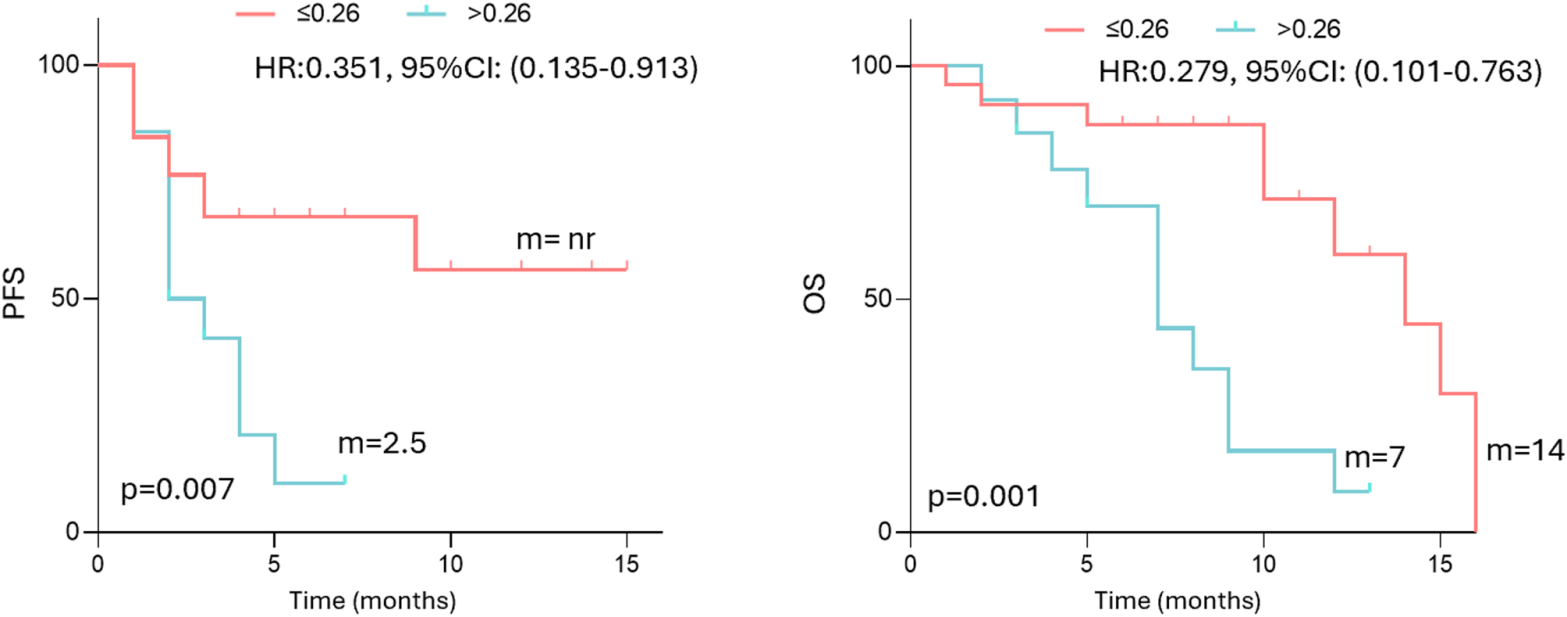
Blood circulating LOX-1⁺PMN-MDSCs subset was validated as a negative prognostic factor of survival in (R/M) HNSCC patients. Kaplan‒Meier curves of PFS and OS calculated in the validation cohort considering the median percentage of LOX-1⁺PMN-MDSCs (0.26%). A long-rank test was used to analyze the differences between the two groups. m = months, nr = not yet reached, p values ≤ 0.05 were considered significant

To validate the role of circulating LOX-1⁺PMN-MDSCs as a negative prognostic factor of survival, we analyzed blood samples obtained from 40 (R/M) HNSCC patients prospectively collected from an independent cohort (see Table 1 for the characteristics of the patients). The UVA confirmed the association of LOX-1⁺PMN-MDSCs (cut-off = 0.26%) with PFS (HR = 0.351, 95% CI (0.135–0.913), *p* = 0.007) and OS (HR = 0.279, 95% CI (0.101–0.763), *p* = 0.001) (Fig. 5), revealing a worse survival outcome for those patients with a percentage of circulating LOX-1⁺PMN-MDSCs > 0.26% before the beginning of immunotherapy. The results validate the prognostic role of LOX-1⁺PMN-MDSCs on survival in (R/M) HNSCC patients.

### Non-responsive patients show a higher increase of LOX-1⁺PMN-MDSCs during pembrolizumab treatment compared to responsive

**Fig. 6:**
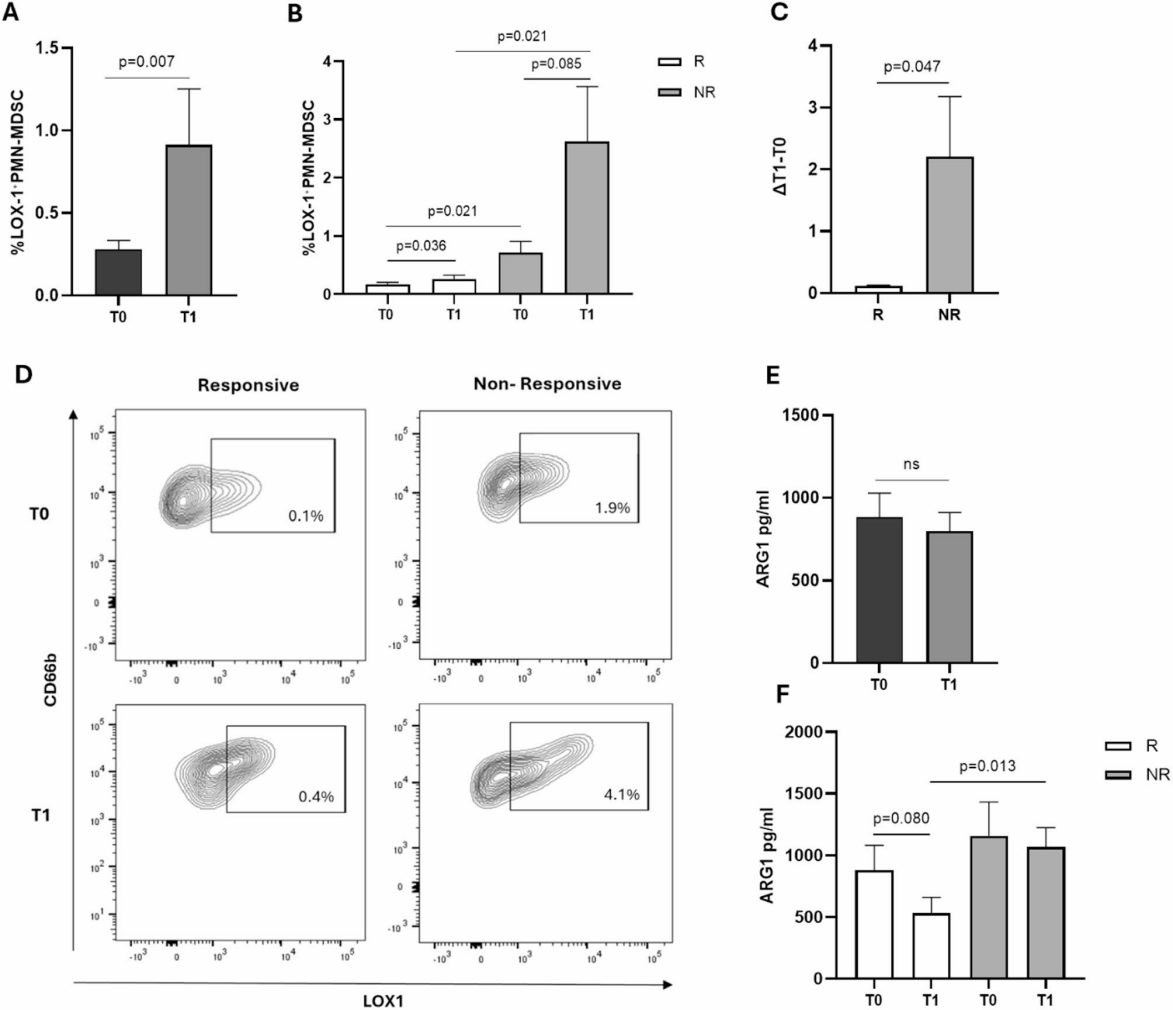
The percentage of LOX-1⁺PMN-MDSCs increased after pembrolizumab treatment in (R/M) HNSCC patients. **A** Results from the analysis of circulating LOX-1⁺PMN-MDSCs in patients with (R/M) HNSCC at baseline (T0) and after one cycle of pembrolizumab treatment (T1) are presented as histograms reporting the means ± SEM. **B** Histograms showing the levels of LOX-1⁺PMN-MDSCs ± SEM in R and NR patients at T0 and T1. **C** Difference between the percentages of LOX-1⁺PMN-MDSCs at T0 and T1 (ΔT1-T0) in R and NR (R/M) HNSCC patients treated with pembrolizumab. p values ≤ 0.05 were considered significant. **D** Cytofluorometric analysis of LOX-1⁺PMN-MDSCs in representative responsive (R) and one non-responsive (NR) (R/M) HNSCC patients at baseline (T0) and after one pembrolizumab cycle (T1). **E** Histograms showing the levels of ARG1, evaluated by ELISA, in the sera of (R/M) HNSCC patients at T0 and T1. **F** Serum levels of ARG1 in R and NR patients at T0 and T1 shown as mean ± SEM. p values ≤ 0.05 were considered significant

To assess whether LOX-1⁺PMN-MDSCs play a role as biomarkers also during immunotherapy treatment, we evaluated their levels in the blood after one pembrolizumab cycle (T1). The results show a significant increase of LOX-1⁺PMN-MDSCs levels during therapy compared to baseline (T0 vs. T1, *p* = 0.007) (Fig. 6A) and this increase was observed in both R and NR patients, although in NR patients the increase was a trend (R: *p* = 0.036; NR: *p* = 0.085) (Fig. 6B). However, the levels of LOX-1⁺PMN-MDSCs were higher in NR vs. R patients at T0 (*p* = 0.021) and remain significantly higher in NRs than in R patients even after one cycle of therapy (T1, *p* = 0.021). This result was also confirmed when considering the difference in circulating LOX-1⁺PMN-MDSCs levels at T1 and T0 (ΔT1-T0); also in this case the increase of LOX-1⁺PMN-MDSCs was significantly more pronounced in NR patients than in R patients (*p* = 0.047) (Fig. 6C). Representative flow cytometry profiles of LOX-1⁺PMN-MDSC in the blood of a responsive and a non-responsive (R/M) HNSCC patient at T0 and T1 are shown in Fig. 6D. Functional activity of LOX-1⁺PMN-MDSCs at T1 again indicated that this subset maintained its suppressive capacity (Additional file 4). This prompted us to investigate whether serum levels of ARG1 could be a marker of their functional state. No significant differences in ARG1 serum levels were observed between T0 and T1 (Fig. 6E). However, when patients were stratified based on response to immunotherapy treatment, it resulted that in R patients ARG1 serum levels decreased after one cycle of therapy compared to T0 (*p* = 0.080), while in NR patients ARG1 levels remained stable at T1, and were significantly higher than ARG1 in R patients (*p* = 0.013, Fig. 6F). These results demonstrate that during treatment R patients are characterized by a significant reduction of circulating levels of ARG1 and that LOX-1⁺PMN-MDSCs may impact the response to pembrolizumab treatment, highlighting their potential role in the clinical outcome of (R/M) HNSCC patients also during immunotherapy.

## Discussion

The suppression of the antitumor immune response is one of the main mechanisms used by tumors to evade immune destruction, and this represents one of the main immunological conditions of the HNSCC tumor microenvironment. Dysregulation of the immune system confers resistance to anticancer therapies and promotes tumor progression. The clinical outcome, which is easily assessed in a limited follow-up due to the immunosuppressive and aggressive nature of this cancer, makes HNSCC an optimal setting for studying the biological mechanisms of immune escape and identifying biomarkers of resistance to ICIs [38, 39]. Accumulating data showed that high levels of immunosuppressive Tregs, MDSCs and tumor-associated macrophages (TAMs) in the TME of HNSCC patients [40, 41] correlate with disease recurrence, decreasing survival [8, 42]. Further investigations of the resistance mechanisms to ICIs and the identification of novel immune biomarkers, that may confer a better clinical outcome, are critical for improving patient selection and treatment efficacy. In this study, we explored the impact of two main immune subsets involved in circulating immune suppression: Treg cells and MDSCs. We selected a cohort of (R/M) HNSCC patients receiving pembrolizumab treatment and the blood levels of immunosuppressive cells were correlated with clinical parameters. For the first time, at our knowledge, we identified and proposed blood circulating immunosuppressive CD137⁺Tregs and LOX-1⁺PMN-MDSCs as immune biomarkers of resistance to ICIs in (R/M) HNSCC patients. The infiltration of Tregs in various human cancers is generally associated with a poor prognosis, as expected from their role in suppressing antitumor immunity. However, several studies demonstrated that high Tregs levels are associated with a favorable prognosis in different cancer settings [43, 44].

Treg cells exert their suppressive function through several mechanisms. For example, they constitutively express CTLA-4, which reduces the expression of CD80/CD86 by antigen-presenting cells (APCs), and the CD25 molecule and require exogenous IL-2, thereby depleting the amount of IL-2 available for T cell activation and proliferation. Treg cells also release immunosuppressive cytokines, such as the inhibitory cytokines IL-10 and TGF-β, which down-regulate the effector mechanisms of the anti-tumor immune response; moreover, they can also convert ATP to adenosine using CD39 and CD73, and secrete granzymes, which can directly kill T cells or APCs [45].

In HNSCC the impact of Tregs as a prognostic factor is still debate. These discrepancies may be attributed to differences in tumor types, molecular characteristics, different distribution of Tregs within the tumor, heterogeneous subsets and markers used to define them [23]. Here we show that among Tregs subsets, only CD137⁺Tregs negatively correlates with clinical outcome in HNSCC patients. The expression of CD137 is tightly regulated and dynamic and its expression is increased following intra-tumoral Treg cell activation [46–49]. The enhanced immunosuppressive capacity of CD137⁺ Tregs appears to be related to the higher expression of Treg-related genes (such as IL15RA, Foxp3, CTLA-4, Helios), the lack of CD127 expression and the demethylation of Treg-specific demethylated region (TSDR) [50]. Several studies show that the presence of CD137⁺Tregs infiltrating murine and human tumors is correlated with poor prognosis [51–53]. Here, we observed that circulating CD137⁺Tregs negatively correlate with the PS and survival of (R/M) HNSCC patients receiving immunotherapy. We showed that patients with PS = 0 had a significantly lower levels of circulating CD137⁺Treg cells, suggesting the critical role of patients’ immunological status in achieving a better clinical outcome and immunotherapy efficacy. Furthermore, patients with lower levels of CD137⁺Treg cells had prolonged survival following immunotherapy administration. Moreover, our functional data confirm that CD137^+^ Tregs, isolated from patient’s blood, exhibit a strong suppressive capacity, releasing higher levels of IL-10 and TGF-β. Therefore, these data suggest that the expression of CD137 promotes Treg suppressiveness, which is necessary for tumor immune escape, preventing the success of immunotherapy. Thus, CD137^+^Tregs could represent a crucial target for the selection of HNSCC patients treated with anti-PD-1 therapy. Future ad hoc studies, in a larger cohort of patients, should be carried out to better define and validate the potential predictive and prognostic value of CD137⁺Treg cells for immunotherapy efficacy as well as to support their employment as a novel target for combined immune treatment. The pivotal role of peripheral immunosuppression in the clinical outcome of (R/M) HNSCC patients was also assessed by circulating LOX-1⁺PMN-MDSCs. During tumor progression, PMN-MDSCs become a prevalent immunosuppressive cell subset in the peripheral blood from where they are recruited to the tumor site to aid in establishing an immunosuppressive milieu that facilitates tumor escape [54, 55]. It has been demonstrated that MDSCs can suppress immune cells through a variety of mechanisms: i.e. recruitment of Treg cells, release of immune suppressive mediators such as IL-10, nitric oxide synthase 2 (NOS2) (specially produced by M-MDSC), ROS and ARG1 (specially produced by PMN-MDSCs) and other inhibitory molecules [56]. These molecules contribute to the suppression of T cell proliferation by inhibiting the expression of the CD3ε chain and inducing T cell apoptosis [56]. In the last years, PMN-MDSCs have been associated with resistance to ICIs therapy in several solid tumors [44, 57–59]. Interestingly, our study showed, for the first time, that the levels of circulating LOX-1⁺PMN-MDSCs represent an independent prognostic factor of poor prognosis in (R/M) HNSCC patients and a lack of benefit from immunotherapy. Subgroup analysis based on treatment regimens (anti-PD-1 as monotherapy vs. chemo-immuno combination) revealed that LOX-1⁺PMN-MDSCs serve as prognostic biomarker in chemo-immunotherapy treated patients, which is the therapy of choice for HNSCC patients. However, chemotherapy does not affect immune cell dynamics during therapy, as shown in Additional file 5.

The impact of these myeloid subsets was also confirmed by the pronounced increase of LOX-1⁺PMN-MDSCs during therapy observed only in NR patients, suggesting that this MDSC population is involved in the resistance to immunotherapy. Functional characterization of LOX-1⁺PMN-MDSCs isolated from (R/M) HNSCC patients revealed their higher immunosuppressive potential compared to the LOX-1⁻ counterpart. Interestingly, ARG1 serum levels were significantly increased in NR patients which display higher levels of LOX-1⁺PMN-MDSCs. Indeed, in our functional settings, circulating LOX-1⁺PMN-MDSCs produced significantly higher levels of ARG1 and ROS than LOX-1⁻PMN-MDSCs, in agreement with other studies [20, 60]. On the other side, M-MDSCs, lower producers of ARG1 in HNSCC settings, did not affect patient prognosis or response, demonstrating that M-MDSCs may not be a prognostic marker in solid tumors [61]. This study is the first to assess the role of LOX-1⁺PMN-MDSCs and CD137⁺Treg cells in the blood of (R/M) HNSCC patients receiving immunotherapy. The results obtained support the hypothesis that targeting immunosuppression in HNSCC patients may increase the response rate to immunotherapy. Strategies combining MDSC-targeted therapy with anti-PD-1 therapy have been investigated in various solid cancers and have shown superior antitumor efficacy [62]. In particular, preclinical studies suggest that the inhibition of MDSCs and the reduction in Tregs enhance immunotherapy in HNSCC [63, 64], supporting the clinical value of CD137⁺Treg and LOX-1⁺PMN-MDSC cells and their target as therapeutic approach. Therefore, focusing on depleting immunosuppression may be one of the major directions of novel promising therapeutic approaches. Moreover, circulating immune biomarkers represent a valid, easy-to-use parameter derived from blood that is non-invasive, easily dosable and available to standard clinical settings providing an evaluable tool for the prolonged monitoring of cancer patients during therapy.

## Conclusions

The results obtained in this work demonstrate the crucial role of circulating immunosuppressive cells in immunotherapy resistance, although validation in a much larger, multicentre, and clinically different patient population is required before they can be considered applicable also to other cancer settings. These cells could serve as useful biomarkers for better patient stratification and assessing therapeutic effectiveness. They are easy to use in clinical practice and may also be used to develop new therapies aimed at improving outcomes for HNSCC patients.

## Supplementary Information

Supplementary Material 1

## Data Availability

Not applicable.

## Abbreviations

APCs: Antigen-presenting cells
ARG1: Arginase 1
CBR: Clinical benefit rate
CFSE: 5,6-carboxyfluorescein diacetate, succinimidyl ester
CPS: Combined positive score
ECOG PS: Eastern Cooperative Oncology Group Performance Status
FCS: Fetal calf serum
FMO: Fluorescence minus one
HNSCC: Head and neck squamous cell carcinoma
HPV: Human papilloma virus
ICIs: Immune checkpoint inhibitors
ICs: Immune cells
IL: Interleukin
LMR: Lymphocyte-to-monocyte ratio
LOX-1: Lectin-type oxidized LDL receptor 1
mAb: Monoclonal antibodies
MDSCs: Myeloid-derived suppressive cells
MFI: Mean fluorescence intensity
M-MDSCs: Monocyte- myeloid-derived suppressive cells
MVA: Multivariate analysis
NK: Natural killer
NOS2: Nitric oxide synthase 2
NR: Non-responsive
NSCLC: Non-small cell lung cancer
ORR: Overall response rate
OS: Overall survival
PBMCs: Peripheral blood mononuclear cells
PD-1: Programmed Cell Death-1
PD-L1: Programmed cell death ligand-1
PD-L2: Programmed cell death ligand-2
PFS: Progression-free survival
PLR: Platelet-to-lymphocyte ratio
PMN-MDSCs: Polymorphonuclear myeloid-derived suppressor cells
R/M: Recurrent/metastatic
R: Responsive
ROS: Reactive oxygen species
RPMI: Roswell Park Memorial Institute Medium
TAMs: Tumor-associated macrophages
TCs: Tumor cells
TGF-β: Transforming growth factor
TIL: Tumor-infiltrating lymphocyte
TMB: Tumor mutational burden
TME: Tumor microenvironment
TNFR: Tumor necrosis factor receptor
Tregs: Regulatory T cells
UVA: Univariate analysis
