# Supplementary material for: Circulating CD137⁺Treg cells and LOX-1⁺PMN-MDSCs as biomarkers of immunotherapy resistance in (R/M) HNSCC patients
Source: J Exp Clin Cancer Res. 2025 Dec 3;44:316. doi: 10.1186/s13046-025-03574-6 (PMC12676864; doi:10.1186/s13046-025-03574-6)

Additional file 1. Regulatory T cells do not affect the performance status or survival of (R/M) HNSCC patients treated with immunotherapy. (A) The percentages of total Tregs ± standard error of the mean (SEM) in patients with PS=0 and PS≥1 are shown as histograms. (B) Kaplan–Meier curves of PFS and OS were generated, and 6.81% was used as the cutoff value for Treg cells at baseline in (R/M) HNSCC patients. m=months

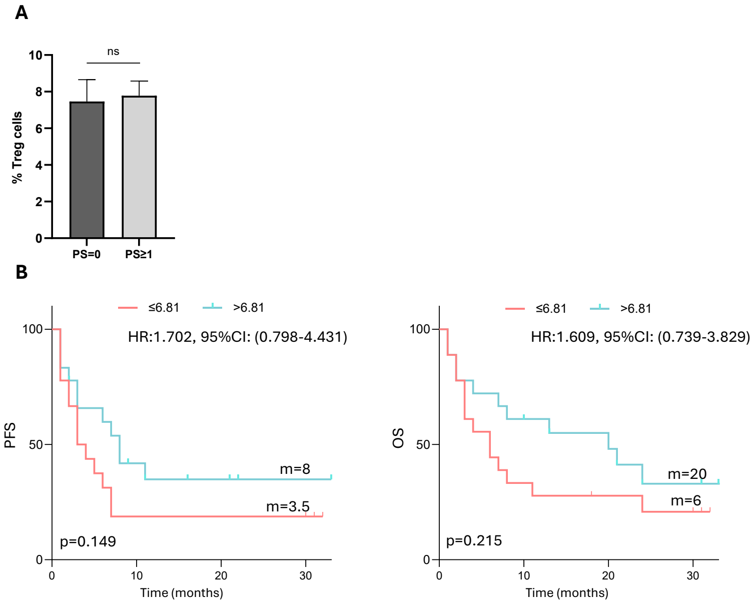

Additional file 2. Intracellular ARG1 and ROS production in M-MDSCs, PMN-MDSCs, and LOX-1 subsets. (A) Histogram showing the MFI ± SEM of ARG1 in M-MDSC, PMN-MDSC, LOX-1⁻ and LOX-1⁺PMN-MDSC. (B) Histogram showing the mean fluorescence intensity (MFI) ± SEM of intracellular ROS levels in M-MDSC, PMN-MDSC, LOX-1⁻ and LOX-1⁺PMN-MDSC detected by DCFDA staining. p values ≤ 0.05 were considered significant.

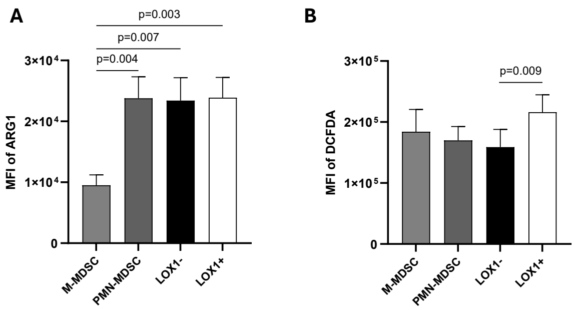

Additional file 3. Performance status is associated with survival in (R/M) HNSCC patients treated with immunotherapy. (A) Kaplan–Meier curves for PFS and OS considering the score related to performance status (PS) (PS = 0 vs. PS≥1). A long-rank test was used to analyze the differences between the two groups. m=months, nr= not yet reached, p values ≤ 0.05 were considered significant.

A

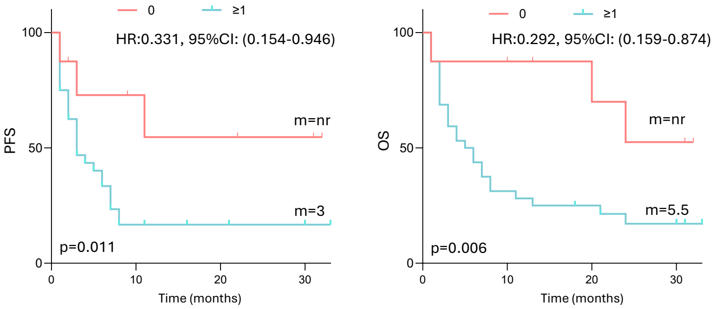

Additional file 4. Immune suppression activity of LOX-1⁺PMN-MDSC derived from peripheral blood of (R/M) HNSCC at T0 and after one pembrolizumab cycle (T1). (A) Suppression assay of LOX-1$^+$PMN-MDSCs, co-cultured for 4 days with autologous T cells, at T0 and T1. The data indicate the mean ± SEM of three independent experiments. (B) Intracellular ROS level in LOX-1$^+$PMN-MDSC detected by DCFDA staining at T0 and T1. (C).Histogram showing the intracellular expression of ARG1 in LOX-1$^+$ PMN-MDSC. p values ≤ 0.05 were considered significant.

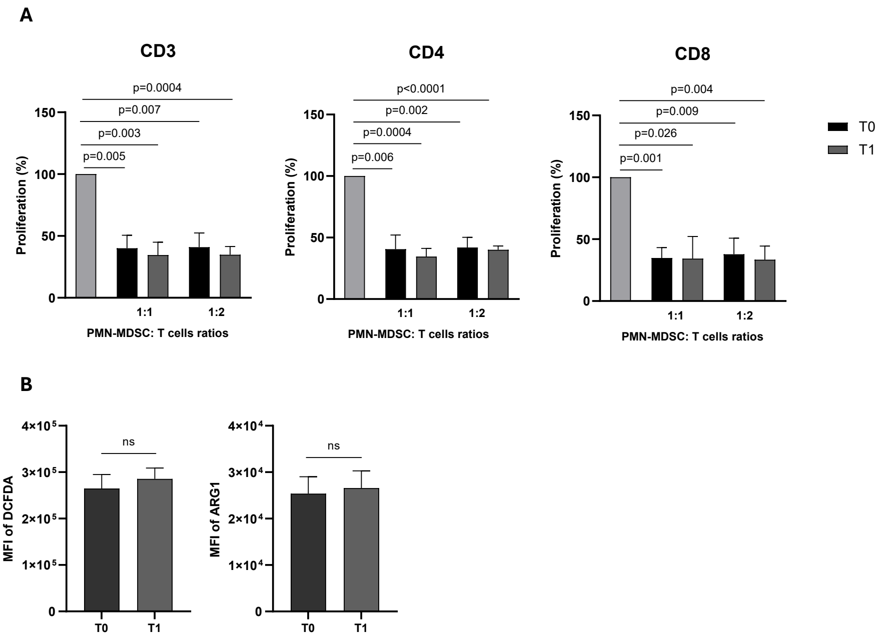

Additional file 5. Prognostic role of LOX-1⁺ PMN-MDSCs in (R/M) HNSCC patients treated with Pembrolizumab alone or in combination with Chemotherapy. (A) Histogram showing the levels of LOX-1^{+}PMN-MDSCs ± SEM in patients received pembrolizumab (Pembro) either as monotherapy or combined with chemotherapy (Pembro+CHT) at T0 and T1. (B) Kaplan–Meier curves for PFS and OS considering the median percentage of LOX-1^{+}PMN-MDSC in Pembro-treated patients at T0. (C) Kaplan–Meier curves of PFS and OS at baseline were generated for patients receiving Pembro+CHT. A long-rank test and the median cut-off value of 0.26% was used to analyze the differences between the two groups. m=months, p values ≤ 0.05 were considered significant.

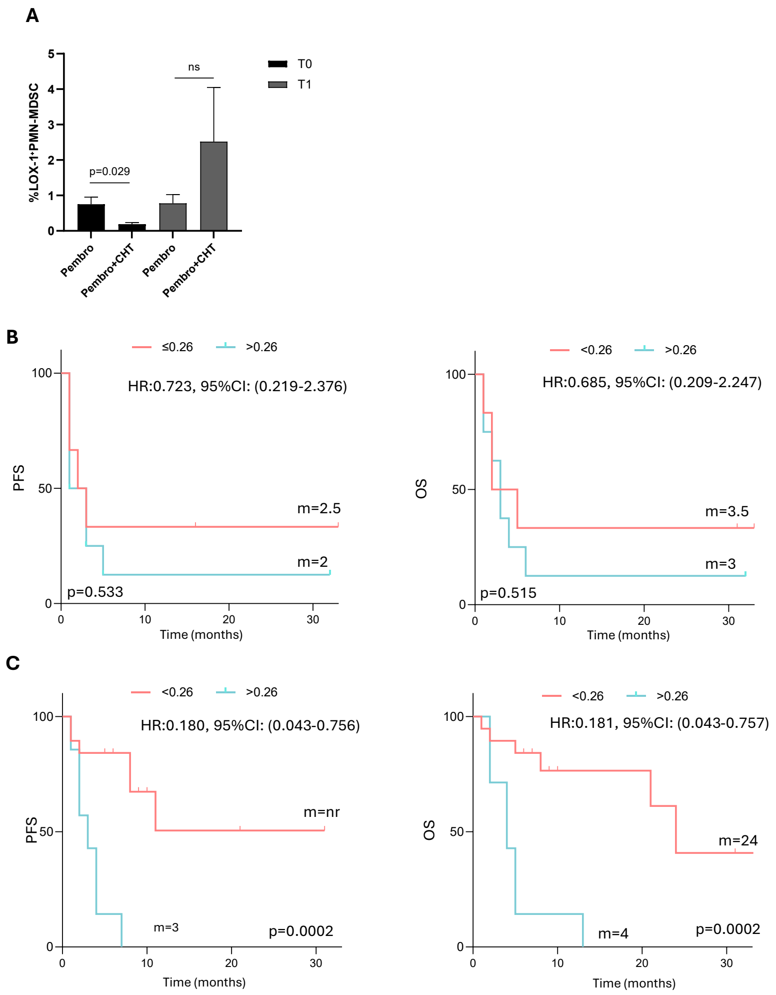

Supplement: Supplementary file 1 — Supplementary Material 1 [file 13046_2025_3574_MOESM1_ESM.pdf]
